# What is the treatment of choice for bulbar urethral strictures which are not an indication for visual internal urethrotomy?

**Published:** 2008

**Authors:** Deepak Dubey, K. Muruganandham

**Affiliations:** Department of Urology, Sanjay Gandhi Postgraduate Institute of Medical Sciences, Lucknow - 226 014, India. E-mail: ddubey@sgpgi.ac.in

## SUMMARY

In this study the authors have retrospectively analyzed the results for 153 patients who underwent end-to-end anastomotic urethroplasty for bulbar urethral strictures of varied etiology. Stricture length was 1 to 2 cm (in 59.5%), 2 to 3 cm (37.9%), 3 to 4 cm (1.9%) or 4 to 5 cm (0.7%). A total of 90 patients (59%) underwent dilation, internal urethrotomy, urethroplasty or multiple procedures before being referred for treatment. Of 153 cases 139 (90.8%) were successful and 14 (9.2%) were treatment failures. Of 14 cases of failure 12 had a satisfactory final outcome after further therapeutic procedures, one is still waiting for the second stage of urethroplasty and one underwent definitive perineostomy. No patients complained of penile chordee or impotence. Bulbar end-to-end anastomosis has a success rate of 90.8%. Most patients were satisfied with the surgical outcome despite postoperative complications such as ejaculatory dysfunction, a glans that was neither full nor swollen during erection, or decreased penile sensitivity.

## COMMENTS

Visual internal urethrotomy is often the first line of management for patients with bulbar urethral strictures. However, the long-term results of this procedure are not more than 30-50%. Its role should be limited for short bulbar urethral strictures (< 1 cm in length) and minimal spongiofibrosis. [1] For all other bulbar strictures, some kind of urethroplasty is generally required. The urethra is the best substitute for itself and whenever possible an end-to-end anastomotic urethroplasty provides the best results for bulbar strictures which are not suitable candidates for visual internal urethrotomy. Performing an anastomotic urethroplasty for strictures of 2-3 cm in length would create a urethral defect of at least 4-5 cm as a 1 cm length of healthy urethra has to be mobilized along the proximal and distal urethra adjacent to the stricture. Some authors have expressed concern that bridging such long defects could lead to chordee and sexual dysfunction and have advocated the use of buccal mucosa substitution urethroplasty for bulbar urethral strictures >2 cm in length. However, in the above study, Barbagli *et al*. [2] managed 57 patients with strictures between 2-3 cm with anastomotic urethroplasty with a long-term success rate of 87%. There was no incidence of chordee in their series. Similarly Elthaway *et al*. [3] reported 98% success rate for bulbar strictures ranging from 0.5-4.0 cm (average 1.9 cm) over a mean follow-up of 50.4 months and Santucci *et al*. [4] reported 95% success rates for excision and anastomosis of bulbar strictures ranging from 0.1-4.5 cm (mean 1.7 cm) over a mean follow-up of 70 months. These studies are important because they report long-term results to the tune of >90%. The verdict is thus clear that excision and anastomosis provides excellent results for bulbar urethral strictures of varied etiology for bulbar stricture lengths averaging 2 cm (range 1-4 cm). They also reinforce the fact that these results can be achieved without having chordee as a complication.
